# Lupus Nephritis Outcomes after Stopping Immunosuppression

**DOI:** 10.3390/jcm13082211

**Published:** 2024-04-11

**Authors:** Fahidah Alenzi, Oier Ateka-Barrutia, Chee Ken Cheah, Munther Khamashta, Shirish R. Sangle, David P. D’Cruz

**Affiliations:** 1 Internal Medicine Department, College of Medicine, Princess Nourah bint Abdulrahman University, Riyadh 11564, Saudi Arabia; 2 Internal Medicine Department, Donostia University Hospital, 20014 Donostia, Spain; oierateka@tutanota.com; 3 Louise Coote Lupus Unit, Counting House, Guy’s Hospital, Guy’s and St. Thomas’ Hospitals, NHS Foundation Trust, London SE1 9RT, UK; cheeken78@gmail.com (C.K.C.); munther.a.khamashta@gsk.com (M.K.); shirish.sangle@gstt.nhs.uk (S.R.S.); david.dcruz@gstt.nhs.uk (D.P.D.)

**Keywords:** lupus nephritis, discontinuation of immunosuppression, systemic lupus erythematosus

## Abstract

**Background/Objectives:** Immunosuppression (IS) is a standard therapy for lupus nephritis (LN). Data on the outcomes of patients with LN after the discontinuation of immunosuppression remain uncertain. This study aimed to evaluate the outcomes and results of patients with lupus nephritis (LN) who ceased immunosuppressive (IS) therapy. **Methods:** Records were obtained on the clinical and laboratory features of LN patients who were treated at our Lupus Unit. They included median values and ranges for various numerical variables such as patient age, disease duration, and treatment duration. Categorical variables such as gender, LN class, IS treatment type, and patient outcomes, which were categorized as either “stable” or “flare experienced”, were presented as percentages and frequencies. A flare in LN was characterized by a two-fold increase in serum creatinine levels and a rise in proteinuria following the cessation of IS medication. **Results:** Outcomes were assessed for 45 patients with LN who ceased IS therapy after achieving remission. The patients’ median age was 55 years (29–78). The median duration of treatment was 4 years (0.5–14). The LN histology distribution was class V = 24.4%, class IV = 17.8 %, class III = 17.8%, class III + IV = 15.6%, class III + V = 6.7%, class IV + V = 2.2%, and class II + IV and II = 2.2%. At the discontinuation of IS treatment, creatinine levels were elevated in 9/45 (20%) patients. Furthermore, 28.9% of patients relapsed after IS treatment discontinuation. Patients with anti-Smith antibodies (anti-Sm) were observed to have a higher occurrence of relapses, with six patients experiencing flare compared to four patients who remained stable (*p* = 0.03). Five (38.5%) of the patients with flares had high creatinine levels after IS discontinuation. **Conclusions:** Most of our patients maintained clinical remission and stable levels of LN parameters after IS treatment discontinuation. Those with a high serum creatinine level, ongoing proteinuria, depleted complement levels, and the presence of anti-Sm antibodies were more likely to experience flares after the discontinuation of IS therapy.

## 1. Introduction

Systemic lupus erythematosus (SLE) is an autoimmune rheumatic disease characterized by the loss of immunological tolerance to endogenous nuclear antigens, resulting in systemic inflammation and damage to various tissues and organs. Most patients with SLE who develop lupus nephritis (LN) do so within 5 years of diagnosis, but it is not uncommon for LN to appear later. In many cases, LN is the first symptom that leads to the diagnosis of SLE [1,2,3]. Women, particularly those of reproductive age, are more likely to develop SLE [4,5]. The overall incidence ranges from 1 to 8 per 100,000 per year [6], with a prevalence of 8 to 180 cases per 100,000 people [4,5]. Some studies have reported significantly higher estimates of the prevalence and incidence of SLE in North America [7]. LN is recognized as a severe manifestation of SLE. Other processes that cause kidney injury in SLE include hypertension, thrombotic microangiopathy, lupus podocytopathy, vascular lesions induced by antiphospholipid antibodies, interstitial tubular nephritis [8], and drug-induced nephrotoxicity, for example, from non-steroidal anti-inflammatory agents and calcineurin inhibitors. Although the prognosis of LN has improved, significant morbidity, partially due to therapy, is common [9,10].

Glucocorticoids and immunosuppressive (IS) therapies such as mycophenolate mofetil (MMF) or cyclophosphamide are standard care therapies for LN. There is widespread agreement that the early detection and prompt treatment of LN exacerbations are critical to achieving renal remission and preventing the development of permanent renal impairment [11]. However, patients with SLE have a lifetime burden of disease, and continued therapy with corticosteroids and IS medication is associated with an increased risk of infection and damage accumulation. Rapid withdrawal from treatment may cause flares of the disease, and there is no consensus on the ideal tapering protocol of IS therapies, leading to discontinuation [12]. Several studies on the discontinuation of IS drugs in LN have found that patients can relapse at any time after achieving the remission and discontinuation of IS drugs, with rates ranging from 4 to 16% per year [13,14,15,16,17,18,19,20]. Treatment guidelines do not provide any advice on the cessation of IS drugs due to a lack of evidence [21,22,23]. To avoid flares, patients with LN in remission are typically kept on IS medication permanently or for prolonged periods [24]. However, long-term exposure to IS medications has been linked to the development of damage in SLE [25]. Therefore, it is necessary to identify successful drug withdrawals. The objective of the current study was to evaluate LN patient outcomes following the cessation of immunosuppression and identify the predictors of successful withdrawal from treatment.

## 2. Materials and Methods

### 2.1. Patients

This was a retrospective study in which the clinical features and laboratory characteristics of patients with LN attending our Louise Coote Lupus Unit were collected. A total of 45 patients with SLE, classified according to the American College of Rheumatology (ACR) criteria, were included; all patients met the ACR classification criteria for LN, and 41 had LN confirmed by biopsy [26]. The patients were treated with glucocorticoids and immunosuppressants, including rituximab, cyclophosphamide, methotrexate, mycophenolate, and/or azathioprine.

### 2.2. Main Outcome Variable

LN flares were defined as any increase in proteinuria above the proteinuria value at the time of IS discontinuation and a doubling of serum creatinine levels. At IS withdrawal, a patient who continued to have no significant proteinuria and whose creatinine level did not double was considered stable. The reasons for stopping IS therapy were achieving remission, conception planning, and patient choice.

### 2.3. Study Factors

Data, including age, ethnicity, sex, duration of disease, duration of follow-up, class of LN, presence of hypertension, lupus autoantibodies, including anti-Smith (anti-Sm) antibodies, double-stranded DNA (anti-dsDNA) antibodies, and antiphospholipid (aPL) antibodies, creatinine level, protein/creatinine ratio, complement C3, complement C4, serum albumin, and previous IS treatments, were collected.

### 2.4. Statistical Analyses

Statistical analyses were performed using IBM SPSS version 27. Numeric response variables, including age, duration of LN, and length of treatment, were described as medians and ranges. Frequencies and percentages were used to present categorical data such as sex, ethnic background, autoantibodies, laboratory characteristics, class of LN, LN medication, and patient outcomes (stable versus flared). The chi-square test was used to compare categorical variables between the patient outcomes. The statistical significance level was set at *p* ≤ 0.05.

## 3. Results

### 3.1. Clinical Outcome after Discontinuation of Immunosuppression

The patients’ median age overall was 55 years (29–78), with the median age of patients with flares being 58 years (41–70), and that of stable patients being 54 years (29–78). Of the 44 women (97.8%), 31 (70.5%) had stable outcomes, while the remaining 13 (29.5%) experienced flare-ups after stopping immunosuppressant therapy. There was only one (2.2%) male patient in our study with a stable outcome. The median duration of the disease was 24 years (15–49) for patients with flares and 27 (15–58) years for stable patients (*p* = ns). Of the 45 patients, 36 had a disease duration of more than 20 years; of these 36 patients, 26 (72.2%) had a stable outcome, and 10 (27.2%) had flares (*p* = 0.947). In total, 10 of the 13 patients with flares (76.9%) had a disease duration of more than 20 years. There was no correlation between total disease duration and the risk of flares after IS discontinuation (r = 0 and *p* = 0.947). Furthermore, 30 of the 45 patients were of Caucasian ethnicity (66.7%); of these, 21 were stable (65.6%), and 9 experienced flares (69.2%). Additionally, 11 of the 45 patients (24.4%) were of African ancestry, with 8 patients identified as having a stable outcome (25%) and 3 with flares (23.1%). Four patients (8.9%) were of Asian ethnicity, with one patient (7.7%) experiencing a flare and three patients (9.4%) having a stable outcome (*p* = 0.970). Following the discontinuation of immunosuppression, the median time to LN flaring was 3 years (1–17), and the median time from IS discontinuation to the last follow-up was 11 years (1–16). Moreover, the mean period of immunosuppression discontinuation and the last follow-up for the patients overall was 9 years (1–16), with stable patients having a mean of 9 years (1–14 years) and patients with flares having a mean of 10 years (3–16) (Table 1).

### 3.2. Lupus Nephritis and Lupus Autoantibodies Parameters, Characteristics, and Outcomes

The LN histological classes were as follows: eleven patients (24.4%) had class V LN, eight patients (17.8%) had class III LN, two patients (4.4%) had class II LN, eight patients (17.8%) had class IV LN, seven patients (15.6%) had combined class III + IV LN, three patients (6.7%) had combined class III + V LN, and one patient each (2.2% each) had class II + IV LN and mixed class IV + V LN. Two patients (4.4%) did not have a biopsy, and two patients (4.4%) had unavailable biopsy results.

Class II LN was identified in two stable patients (4.4%), while class III LN was found in four patients with flares (8.9%) and four stable patients (8.9%). Furthermore, class IV LN was found in one patient with a flare (2.2%) and seven stable patients (15.6%), while class V LN was found in four patients with flares (8.9%) and seven stable patients (15.6%). Furthermore, one patient with a flare (2.2%) and six stable patients (13.3%) had combined class III + IV LN, whereas one patient with a flare (2.2%) had combined class IV + V LN. In addition, one stable patient (2.2%) had class II + IV LN, whereas two stable patients (4.4%) and one patient with a flare (2.2%) had combined III + V LN. No data were available for LN histopathology in four patients (8.9%) (Table 2). At IS treatment discontinuation, creatinine levels were elevated in 9/45 (20%) patients, and the median creatinine level was 73 µmol/L (41–117). Upon the discontinuation of IS treatment, five patients with flares (38.5%) had high creatinine levels and eight (61.5%) had normal serum creatinine, with a median creatinine value of 78 µmol/L (41–111). Among 32 stable patients, 4 (12.5%) had abnormal creatinine values and 28 (87.5%) had normal creatinine levels (*p* = 0.048). Furthermore, the urine-protein-to-creatinine ratio was significant in eight LN patients with flares (88.9%) and one patient (11.1%) with a stable outcome (*p* < 0.001). Of all patients, 12 were found to have low levels of complement C3. Among these, seven (58.3 %) experienced a disease flare, while five (41.7%) remained stable (*p* = 0.009). In addition, 8 of the 12 patients with low complement C4 had flares (66.7%), compared to 4 who had stable outcomes (33.3%; *p* = 0.001). Furthermore, 20 patients (45%) had low serum albumin levels < 40 g/L, where 9 of them (45%) had a flared outcome and 11 (55%) had a stable outcome (*p* = 0.033; Table 1 and Table 3).

Patients with anti-Sm antibodies were observed to have a higher occurrence of relapses, with six patients experiencing flares compared to four patients who remained stable (*p* = 0.03). Of the 32 stable patients, 22 showed the presence of anti-*dsDNA* antibodies (68.8%), while 10 out of 13 relapsed patients (31.3%) tested positive for these antibodies (*p* = 0.57).

Of the 32 stable patients, 14 (66.7%) tested positive for anti-Ro antibodies, while 7 of the 13 relapsed patients (33.3%) showed positive results (*p* = 0.594). Of the total, 22 patients tested negative for anti-Ro antibodies, with 17 (77.3%) having a stable outcome and 5 (22.7%) having a flared outcome. The anti-Ro antibody result was not available for two patients, one with a stable outcome and the other one with a flared outcome. In total, 4 (25%) of all patients had anti-La antibodies, 1 with a flare and 3 (75%) with stable outcomes, compared to 39 patients who had a negative anti-La antibody result, with 28 (71.8%) having a stable outcome and 11 (28.2%) having flares (*p* = 0.795). Among the 13 flared patients, only 1 had a positive anti-La test (7.7%), whereas 11 (84.6%) were negative for anti-La antibodies, and data were unavailable for 1 patient. In addition, anti-ribonuclear protein (RNP) antibodies were found in 10 of the 45 patients: 5 (50%) had a stable result and 5 (50%) had a flared outcome, while of the 32 (71.1%) patients who had negative anti-RNP antibody results, 25 (78.1%) had a stable outcome and 7 (21.9%) had a flared outcome (*p* = 0.227). Three patients had no anti-RNP antibody data; of these, two patients (66.7%) had a stable outcome, while one patient (33.3%) had a flared outcome. Of the 13 patients diagnosed with antiphospholipid syndrome (APS), 10 (76.9%) had a stable outcome and 3 (23.1%) had flares (*p* = 0.84). A total of 3 (23%) of the 13 patients with flares had APS syndrome, 5 (38.5%) had antiphospholipid antibody (aPL) antibodies, and 5 (38.5%) had negative results for aPL antibodies (Table 4).

### 3.3. Previous Immunosuppressive Medications

Of the 45 patients, 13 (28.9%) previously received azathioprine (AZA) treatment, 10 (22.2%) received the Euro-lupus regimen of intravenous (IV) cyclophosphamide followed by azathioprine, 7 (15.6%) received IV cyclophosphamide, 3 (6.7%) received IV cyclophosphamide followed by mycophenolate, 3 (6.7%) received mycophenolate, 3 (6.7%) received mycophenolate and azathioprine, 2 (4.4%) received IV cyclophosphamide followed by methotrexate(MTX), 1 (2.2%) received azathioprine and methotrexate, and 1 (2.2%) received mycophenolate followed by azathioprine. Thirteen patients relapsed after receiving IS therapy in the following manner: four patients (30.8%) received AZA, three patients (23.1%) received IV cyclophosphamide followed by mycophenolate, two patients (15.4%) received IV cyclophosphamide, and two patients (15.4%) received mycophenolate. IS treatment data for two patients (15.4%) were unavailable (Table 5). Furthermore, seven patients discontinued hydroxychloroquine and had stable outcomes. The 13 patients who flared up were treated with hydroxychloroquine.

## 4. Discussion

Despite conventional IS treatments, renal relapses occur in 15–43% of patients after 3 years, and 5–20% of patients progress to end-stage renal disease after 10 years. However, drug-induced toxicity remains an issue [27,28]. The appropriate duration of IS treatment for LN is uncertain, and it is still debatable whether and when IS drug maintenance therapy can be stopped in LN once remission is achieved. While reducing or discontinuing IS treatment before 18 months seems to be associated with a significant risk of recurrence and subsequent organ damage, the long-term maintenance of IS can be associated with a greater incidence of adverse events, such as cardiovascular events, infections, and malignancies [9,29]. Remission can be regarded as a treatment goal in managing LN and is associated with a better prognosis [30,31,32,33,34,35]. However, it is still unclear how to manage patients in remission [36,37,38,39]. Treatments for patients with SLE in clinical remission should seek to prevent disease flares while avoiding overtreatment and reducing damage accumulation [18]. There is little evidence that treating patients with lupus who have achieved clinical and serological remission with prolonged immunosuppression for many years improves their long-term prognosis [18]. In this setting, there are limited data on IS drug withdrawal, and only a few studies have reported the outcomes of the withdrawal of medications in patients with LN [23].

We assessed 45 patients with LN who discontinued IS medication. In total, 13 of the 45 (28.9%) patients relapsed after the discontinuation of IS treatment. Similarly, Pablos et al. [18] investigated the discontinuation of cyclophosphamide after 2 years of complete renal remission in 11 patients with class IV LN and found that 36% of the patients relapsed.

The plasma creatinine concentration doubled in LN patients who stopped treatment, according to a study by Moroni et al. [12], compared to patients who continued therapy, which is similar to our data. In contrast, a study by Zen et al. [40] reported that patients in prolonged remission and those with flares following IS treatment cessation had similar serum creatinine levels after the study. Our observations revealed that the median age of patients when they experienced a flare-up was 58 years (41–70), and the median time to experience an LN flare-up was 3 years (1–17). Moroni et al. [17] described 52 LN patients who discontinued IS medications over a median follow-up of 101.8 months: 32 patients (61.3%) did not develop any flare after treatment withdrawal, and patients who received LN therapy for a longer period and experienced remission before the withdrawal of IS treatment and staying on hydroxychloroquine did not flare up [17]. Mosca et al. stopped cyclophosphamide in 33 LN patients, and following the termination of cyclophosphamide, 15 patients (45%) suffered a renal flare; 24% of these flares occurred within 2 years after medication termination (early flares), and the remaining 21% occurred more than 2 years later [16].

In this study, positive anti-dsDNA antibodies were more prevalent in stable patients than in patients with relapse. Zen et al. [40] concluded that anti-dsDNA antibody levels were similar in patients with and without flares after IS discontinuation, with anti-dsDNA antibodies being present in most patients. Furthermore, as shown in previous studies, anti-dsDNA antibodies were linked to renal disease in SLE patients, and patients [41] who are at a higher risk of lupus flare-up could be identified [42]. Moreover, previous studies [17,40] found no difference in the risk of relapse between patients with low or normal complement C3 and C4 levels.

According to our study, patients who stopped azathioprine were more likely to experience flares than those who stopped taking other IS drugs. A higher percentage of patients who had previously received azathioprine experienced a flared relapse compared to those who had previously used cyclophosphamide or MMF (30.8% vs. 15.4%, respectively). A study by Zen et al. [43] reported that, compared to those who stopped using other IS drugs, patients who stopped taking MTX were more likely to flare, and there was no statistical difference between the groups with and without flares who had stopped using AZA and MMF. Furthermore, there was no difference in the rate of lupus relapse between patients who discontinued MMF and those who continued to receive MMF in a 60-week randomized, unblinded study, in which 76% of the patients had a history of LN [44]. In addition, Chakravarty et al. reported that MMF maintenance was associated with higher rates of adverse effects and infections compared to discontinuation [23]. The limitations of this study include a retrospective nature with a small sample size and a risk of missing data. No data on medication non-adherence were collected.

## 5. Conclusions

Most of our patients maintained clinical remission and stable levels of LN parameters after IS treatment discontinuation. Those with high serum creatinine levels, ongoing proteinuria, depleted complement levels, and the presence of anti-Sm antibodies were more likely to experience a flare after the discontinuation of IS therapy.

Further prospective studies with longer follow-up periods and larger sample sizes are needed to estimate LN outcomes after immunosuppression discontinuation. A randomized trial of treatment withdrawal versus continued treatment in LN would be ideal.

## Figures and Tables

**Table 1:** Patients’ characteristics and lupus nephritis outcome.

Parameters n (%)	Flared Patients (N = 13)	Stable Patients (N = 32)	*p* Value
Median age, year	58	54	0.504
Gender, Female n (%)	13 (29.5%)	31 (70.5%)	0.519
Median duration of the disease, years	24	27	0.947
Ethnicity n (%) Caucasian Black African Asian	9 (69.2%) 3 (23.1%) 1 (7.7%)	21 (65.6%) 8 (25%) 3 (9.4%)	0.970
Abnormal creatinine n (%)	5 (55.6%)	4 (44.4%)	0.048
Significant UPCR n (%)	8 (88.9%)	1 (11.1)	0.000
Low albumin n (%)	9 (45%)	11 (55%).	0.033
Low C3 n (%) Low C4 n (%)	7 (58.3%) 8 (66.7%)	5 (41.7%) 4 (33.3%)	0.009 0.001

UPCR—urine protein creatinine ratio, C3—complement 3, and C4—complement 4.

**Table 2:** Lupus nephritis class and patient outcome.

LN Class	Flared Patients (N = 13)	Stable Patients (N = 32)
Class II	-	2 (4.4%)
Class III	4 (8.9%)	4 (8.9%)
Class IV	1 (2.2%)	7 (15.6%)
Class V	4 (8.9%)	7 (15.6%)
Class III + IV	1 (2.2%)	6 (13.3%)
Class IV + V	1 (2.2%)	-
Class II + IV	-	1 (2.2%)
Class III + V	1 (2.2%)	2 (4.4%)
No data were available for LN histopathology	1 (2.2%)	3 (6.7%)

**Table 3:** Lupus nephritis parameters and characteristics.

At Discontinuation of IS		After Discontinuation of IS	
Total Patients (n = 45)		Flared (n = 13)	
9/45 (20%) had elevated serum creatinine level	Median creatinine value was 73 (41–117) umol/L	5/13 (38.5%) had high serum creatinine level	Median creatinine values were 78 (41–111) umol/L
12 patients had low C3 level; 7/12 (58.3%) were flared and 5/12 (41.7%) were stable (*p* = 0.009)	8/12 patients with low C4 flared (66.7%), compared to 4/12 who remained stable (33.3%) (*p* = 0.001).	8/13 (88.9%) had elevated UPCR; 1 patient (11.1%) had a stable outcome (*p* < 0.001).	20/45(45%) had low serum albumin levels (<40 g/L), of whom 9 (45%) had flared and 11 (55%) remained stable (*p* = 0.033).

IS—immunosuppression, UPCR—urine protein creatinine ratio, C3—complement 3, and C4— complement 4.

**Table 4:** Patient autoantibodies characteristics and lupus nephritis outcome.

Autoantibody (+) n (%)	Flared Patients (N = 13)	Stable Patients (N = 32)	*p* Value
Anti-Sm (+) n (%)	6 (46.2%)	4 (12.5%)	0.030
Anti-dsDNA (+) n (%)	10 (31.3%)	22 (68.8)	0.567
Anti-Ro (+) n (%)	7 (33.3%)	14 (66.7%)	0.594
Anti-La (+) n (%)	1 (25%)	3 (75%)	0.790
Anti-RNP (+) n (%)	5 (50%)	5 (50%)	0.227
aPL (+) n (%) APS (+) n (%)	5 (33.3%) 3 (23.1%)	10 (66.7%) 10 (76.9%)	0.835

Anti-Sm—anti-Smith antibody, Anti-dsDNA—anti-double-stranded DNA antibodies, Anti-Ro— autoantibodies directed against Ro/SSA antigens, Anti-La—autoantibodies directed against La/SBB antigens, Anti-RNP—antinuclear ribonucleoprotein antibody, aPL—antiphospholipid antibodies, and APS—antiphospholipid syndrome.

**Table 5:** Type of immunosuppression (IS) used for lupus nephritis.

IS Used	Flared Patient (N = 13)	Stable Patient (N = 32)
AZA	4 (30.8%)	9 (69.2%)
Cyclo	2 (28.6%)	5 (71.4%)
MMF	2 (66.7%)	1 (33.3%)
Cyclo followed by AZA	0 (0%)	10 (100%)
Cyclo followed by MMF	3 (100%)	0 (0%)
MMF followed by AZA	0 (0%)	3 (100%)
MMF followed by MTX	0 (0%)	1 (100%)
Cyclo followed by MTX	0 (0%)	2 (100%)
AZA + MTX	0 (0%)	1 (100%)
NA	2 (100%)	0 (0%)

AZA—azathioprine, Cyclo—cyclophosphamide, MMF—mycophenolate, MTX—methotrexate, and NA—not available.

## Data Availability

The study materials and datasets are available from the corresponding author upon reasonable request.
